# Association between inflammatory markers and non-alcoholic fatty liver disease in obese children

**DOI:** 10.3389/fpubh.2022.991393

**Published:** 2022-12-01

**Authors:** Yamei Duan, Jiayou Luo, Xiongfeng Pan, Jia Wei, Xiang Xiao, Jingya Li, Miyang Luo

**Affiliations:** ^1^Department of Maternal and Child Health, Xiangya School of Public Health, Central South University, Changsha, Hunan, China; ^2^Department of Epidemiology and Health Statistics, Xiangya School of Public Health, Central South University, Changsha, Hunan, China

**Keywords:** inflammatory markers, cytokines, non-alcoholic fatty liver disease, association, obesity children

## Abstract

**Background:**

Given the high prevalence of non-alcoholic fatty liver disease (NAFLD) in obese children, non-invasive markers of disease to date are still limited and worth exploring.

**Objective:**

This study aimed to evaluate the association between inflammatory markers and NAFLD in obese children.

**Methods:**

We performed a case-control study in Hunan Children's Hospital from September 2020 to September 2021. Study participants were children with obesity diagnosed with NAFLD by abdominal ultrasound examination. Mean platelet volume (MPV), platelet distribution width (PDW), neutrophil, lymphocyte, monocyte, and platelet counts were extracted from medical records and inflammatory cytokines were measured by enzyme-linked immunosorbent assay (ELISA). Multivariable logistic regression analysis was performed to evaluate the association between inflammatory markers and NAFLD. We also used receiver operating characteristic curve analysis to assess the discriminative ability of inflammatory cytokines for NAFLD.

**Results:**

Two hundred and sixty-seven obese children were enrolled, including 176 NAFLD patients and 91 simple obesity controls. Multivariable logistic model indicated that increased interleukin (IL)-1β [odds ratio (OR) = 1.15, 95% confidence interval (CI): 1.04–1.27], IL-6 (OR = 1.28, 95% CI: 1.07–1.53), and IL-17 (OR = 1.04, 95% CI: 1.02–1.07) levels were significantly associated with NAFLD. In contrast, we observed non-significant associations for IL-8, IL-12, IL-21, IL-32, tumor necrosis factor-α (TNF-α), neutrophil to lymphocyte ratio (NLR), platelet to lymphocyte ratio (PLR), lymphocyte to monocyte ratio (LMR), mean platelet volume (MPV), and platelet distribution width (PDW) with NAFLD. The area under the curves (AUCs) of IL-1β, IL-6, and IL-17 to discriminate obese children with or without NAFLD were 0.94, 0.94, and 0.97, respectively.

**Conclusions:**

Our results indicated that IL-1β, IL-6, and IL-17 levels were significantly associated with NAFLD. These inflammatory cytokines may serve as non-invasive markers to determine the development of NAFLD and potentially identify additional avenues for therapeutic intervention.

## Introduction

Non-alcoholic fatty liver disease (NAFLD) has become increasingly prevalent among children with the epidemic of overweight and obesity in children (1–3). NAFLD has become the most common chronic liver disease worldwide, with a global prevalence of 20 to 50% in obese children (4–6). NAFLD is currently the fastest growing cause of liver-related mortality and is becoming a significant cause of end-stage liver disease, primary liver cancer, and liver transplantation, creating huge health and economic burden (7).

NAFLD encompasses a spectrum of liver pathologies, including non-alcoholic fatty liver (NAFL), non-alcoholic steatohepatitis (NASH), fibrosis, and hepatocellular carcinoma (8, 9). Although the pathogenesis of NAFLD has not been fully identified, the “multiple-hit” hypothesis is now widely accepted. With this hypothesis, lipid accumulation triggers steatosis, which subsequently causes multiple injuries and may eventually lead to NASH and cirrhosis (10–12), involving multiple mechanisms such as insulin resistance (13), abnormal lipid metabolism (14), inflammatory response (15), genetic polymorphism and epigenetics (16). In particular, disorders of lipid metabolism and its secondary cellular inflammatory response on this basis are thought to be important mechanisms in the pathogenesis of NAFLD (17).

Increasing attention has been paid to the involvement of inflammatory markers in the inflammatory response, including inflammatory cytokines, inflammatory cells, and platelets (7, 18). It has been shown that inflammatory cytokines were essential factors contributing to NAFLD and could be used as biomarkers to evaluate the development of disease and predict prognosis, such as interleukin (IL)-1β, IL-6, tumor necrosis factor-α (TNF-α) (19, 20). In addition, various studies have reported elevated levels of pro-inflammatory cytokines in NAFLD patients (20, 21). Similar to inflammatory cytokines, neutrophil to lymphocyte ratio (NLR), platelet to lymphocyte ratio (PLR), lymphocyte to monocyte ratio (LMR), mean platelet volume (MPV), and platelet distribution width (PDW) have recently been used as potential novel biomarkers of inflammatory progress (22, 23). A study found that NLR was elevated in advanced inflammation, which was useful in assessing the extent of disease in patients with NAFLD (24). Another study found that platelets played an essential role in the spread and initiation of inflammatory diseases (25). And MPV and PDW could be considered as markers of platelet activation and function (23). However, the relationship between these inflammatory markers and NAFLD remains controversial. Some studies showed positive associations between inflammatory markers and NAFLD (26, 27), while other studies showed negative or null associations (28, 29). Notably, there is a lack of data in the literature about the association of PLR and MPV with NAFLD.

In this study, we aimed to evaluate the association between inflammatory markers and NAFLD in obese children, including IL-1β, IL-6, IL-8, IL-12, IL-17, IL-21, IL-32, TNF-α, NLR, PLR, LMR, PDW, and MPV. Furthermore, we planned to explore the utility of these inflammatory markers in discriminating obese children with or without NAFLD in order to identify biomarkers for the development of NAFLD in obese children.

## Methods

### Study design and participants

We performed a case-control study in Changsha, China, with a sample of 267 obese children, who attended to the Hunan Children's Hospital between September 2020 and September 2021 for management of obesity. Participants of both sexes aged between 7 and 17 years were selected. Obesity was defined based on the body mass index-for-age (BMI-for-age, z-score) indicator, using the “Classification Criteria for Overweight and Obesity Screening Body Mass Index Values for Chinese School-age Children and Adolescents” recommended by the China Obesity Task Force. The study protocol was approved by the Ethics Committee of the Xiangya School of Public Health in accordance with the principles of the Declaration of Helsinki. All study participants signed an informed consent form before the start of the study.

The following inclusion criteria of participants were included: (a) the patients were diagnosed with NAFLD; (b) the children and guardians agreed to participate in the study and signed an informed consent form; (c) relevant examinations were completed; (d) questionnaires were completed and qualified. Exclusion criteria were as follows: (a) secondary obesity in children; (b) children were taking lipid-lowering medication; (c) suffered from severe cognitive impairment and were unable to communicate in words or language; (d) no informed consent form signed; (e) relevant examinations were not completed; (f) questionnaires were not completed and qualified.

### NAFLD diagnosis

NAFLD was diagnosed by abdominal ultrasound according to the guidelines for the diagnosis and management of NAFLD by the Chinese Society of Hepatology (2018) (30). The diagnosis of NAFLD was required for the following three conditions: (a) no history of alcohol consumption or consumption of < 30 g/d of alcohol equivalent for men and < 20 g/d for women. (b) The diagnosis of fatty liver was based on diffusely enhanced echogenicity in the anterior field of the liver, i.e., bright liver, with stronger echogenicity than in the kidney, poor visualization of intrahepatic ductal structures, and gradual attenuation of echogenicity in the far field of the liver. (c) excluded specific liver diseases that can cause fatty liver such as alcoholic fatty liver, chronic hepatitis C virus infection, autoimmune hepatitis, and hepatomegaly, and excluded specific conditions that cause fatty liver such as drugs, total parenteral nutrition, inflammatory bowel disease, celiac disease, hypothyroidism, Cushing syndrome, β lipoprotein deficiency, α-1 antitrypsin deficiency, and certain insulin resistance-related syndromes.

Children with obesity were subclassified into three groups based on the severity of NAFLD: Simple obesity was defined as no NAFLD on imaging and normal liver function; NAFL was defined as NAFLD on imaging but normal liver function; and NASH was defined as NAFLD on imaging and liver function injury. The diagnostic criteria for liver function injury were alanine aminotransferase (ALT) > 60 IU/L.

### Clinical and laboratory assessment

Demographic data were obtained through structured interviews and questionnaires. Height, weight, waist, and hip circumference were measured two times while standing and wearing light clothing, and average values were used for analysis. Body mass index (BMI) was calculated as the weight divided by the square of the height.

Fasting whole blood samples were collected *via* venipuncture in the morning after a fasting period of longer than 8 h. Laboratory assays were performed in hospital laboratory departments, which included white blood cell count (WBC), hematocrit, PDW, MPV, neutrophil count, lymphocyte count, monocyte count, total bilirubin, ALT, aspartate aminotransferase (AST), albumin, triglycerides (TG), total cholesterol (TC), glycated hemoglobin A1C (HbA1c), high-density lipoprotein cholesterol (HDL-C), low-density lipoprotein cholesterol (LDL-C), fasting blood glucose (mmol/L, FBG), and fasting insulin (μU/ml, FINS). The homeostasis model assessment of insulin resistance (HOMA-IR) was calculated using the formula: [fasting insulin (μU/ml) ^*^ fasting glucose (mmol/L)/22.5]. The NLR was calculated as neutrophil count divided by lymphocyte count. The PLR was calculated as platelet count divided by lymphocyte count. The LMR was calculated as lymphocyte count divided by monocyte count.

Peripheral venous blood samples were collected from all subjects using an ethylenediamine tetraacetic acid (EDTA) anticoagulation tube. Blood samples were centrifuged at 3,500 r/min for 15 min using a low-speed centrifuge. Two aliquots of plasma and blood cells were separated, then labeled and stored at −80°C until the determination. Plasma levels of IL-1β, IL-6, IL-8, IL-12, IL-17, IL-21, IL-32, and TNF-α were measured by an enzyme-linked immunosorbent assay (ELISA) using kits supplied by Hunan Saiwang Biotechnology Co., LTD (Renjiebio, China kits).

### Statistical analysis

Continuous variables were described as means ± standard deviation (SD) or medians (interquartile range, IQR). Different comparative analyses were performed using Student's *t*-test or the Mann-Whitney U test for continuous variables, and Pearson's chi-square test for categorical variables. The profile of correlations between biochemical parameters was examined by network analysis of the Spearman's correlation matrices. Correlations with *P* < 0.05 were included in the network visualization. Spearman's rank values (rho) were used to describe the strength of correlations between NAFLD and concentrations of circulating biochemical parameters. Univariable logistic regression analysis and multivariable logistic regression analysis with stepwise selection were constructed to estimate the odds ratios (ORs) and 95% confidence intervals (CIs) of associations between inflammatory markers and NAFLD, including NAFL and NASH. Multivariable logistic regression analysis was adjusted for all potential confounders with statistically significant associations in comparison of baseline demographics and clinical characteristics between two groups. Receiver operating characteristic (ROC) curve analysis was used to estimate the performance of plasma biomarker levels in distinguishing obese children with NAFLD from simple obese children. All comparisons were two-tailed and *P* < 0.05 were statistically significant. The statistical analyses were performed using SPSS version 20.0 software (SPSS Inc., Chicago, IL, USA).

## Results

### Characteristics of the study population

From September 2020 to September 2021, a total of 267 participants were enrolled, of which 176 obese children were diagnosed with NAFLD and 91 were simple obese controls. Table 1 shows the clinical characteristics and biochemical measurements of the population studied. Levels of age, HbA1c, TG, AST, and ALT were significantly higher in the NAFLD group than in the simple obesity group (all *P* < 0.05). Moreover, the level of HDL-C was significantly lower in the NAFLD group than in the simple obesity group (*P* = 0.004). In terms of sex, BMI, WHR, SBP, DBP, FBG, FINS, HOMA-IR, TC, LDL-C, total bilirubin, WBC, hematocrit, and albumin, no significant differences were found between the two groups (all *P* > 0.05).

**Table 1:** Demographics and clinical characteristics of study participants.

**Variables**	**Simple obesity (*n* = 91)**	**NAFLD (*n* = 176)**	***P*-value**
Sex (male/female)	75/16	150/26	0.550
Age (years)	10.34 ± 1.94	10.84 ± 1.88	**0.045**
BMI (kg/m^2^)	27.84 ± 3.06	28.32 ± 3.84	0.308
WHR	0.83(0.79–0.86)	0.84(0.79–0.90)	0.138
SBP (mmHg)	117.00(111.00–129.00)	120.00(110.00–129.00)	0.456
DBP (mmHg)	70.00(64.00–78.00)	70.00(65.00–77.00)	0.973
FBG (mmol/L)	4.50(4.19–4.78)	4.51(4.18–4.80)	0.799
FINS (μU/ml)	21.56(15.23–29.71)	24.95(15.30–39.17)	0.061
HOMA-IR	4.15(2.92–6.18)	4.85(2.92–8.24)	0.060
HbA1c (%)	4.75(4.44–5.09)	4.49(0.40–4.94)	**0.002**
TG (mg/dl)	1.12(0.80–1.52)	1.37(0.94–1.93)	**0.002**
TC (mg/dl)	3.97 ± 0.80	4.03 ± 0.76	0.529
HDL-C (mg/dl)	1.20 ± 0.24	1.12 ± 0.23	**0.004**
LDL-C (mg/dl)	2.20 ± 0.70	2.32 ± 0.65	0.174
AST (U/L)	21.2(17.8–26.6)	29.80(24.03–45.90)	**< 0.001**
ALT (U/L)	21.0(16.6–29.9)	49.45(28.73–82.80)	**< 0.001**
Total bilirubin (μmol/L)	9.30(7.20–12.70)	10.40(8.23–12.75)	0.080
WBC (10^9^ /L)	7.76 ± 1.89	7.90 ± 1.87	0.586
Hematocrit (%)	40.50 ± 5.01	41.62 ± 3.13	0.053
Albumin (g/L)	42.06 ± 2.44	42.50 ± 2.90	0.193

Data are expressed as means ± SD or medians (IQR).

The bold values mean the *P*-values with statistically significant (*P* < 0.05).

BMI, body mass index; WHR, waist circumference to hip circumference ratio; SBP, systolic blood pressure; DBP, diastolic blood pressure; TG, triglycerides; TC, total cholesterol; FBG, fasting blood glucose; FINS, fasting insulin; HbA1c, glycosylated hemoglobin; HOMA-IR, homeostasis model assessment of insulin resistance; HDL-C, high-density lipoprotein cholesterol; LDL-C, low-density lipoprotein cholesterol; AST, aspartate aminotransferase; ALT, alanine aminotransferase; WBC, white blood cell count.

### Circulating levels of inflammatory markers by NAFLD diagnosis

We compared the levels of NLR, PLR, LMR, PDW, MPV, IL-1β, IL-6, IL-8, IL-12, IL-17, IL-21, IL-32, and TNF-α in simple obese children and obese children with NAFLD. Analyses of the circulating levels of inflammatory markers in obese children revealed that circulating IL-1β, IL-6, IL-8, IL-12, IL-17, IL-21, IL-32, and TNF-α levels were differentially expressed in plasma samples. Specifically, we observed the levels of IL-1β, IL-6, IL-8, IL-12, IL-17, IL-21, IL-32, and TNF-α were significantly higher in the NAFLD group than in the simple obesity group (all *P* < 0.001). Interestingly, we observed an increasing presentation among the simple obesity group, the NAFL group, and the NASH group. The lowest levels of inflammatory cytokines were found in the simple obesity group, followed by the NAFL group, and the highest in the NASH group, with a statistically significant difference among the three groups. In addition, our results indicated the levels of NLR, PLR, LMR, PDW, and MPV were higher in the NAFLD group compared to the simple obesity group. However, no significant differences were found in the levels of these inflammatory markers between the two groups (all *P* > 0.05) (Table 2; Supplementary Table 1).

**Table 2:** Levels of inflammatory markers of the subgroup by NAFLD diagnosis.

**Variables**	**Simple obesity (*n* = 91)**	**NAFLD (*n* = 176)**	***P*-value**
NLR	1.42(1.17–1.96)	1.50(1.15–1.83)	0.589
PLR	117.79(95.96–151.17)	113.99(93.73–136.27)	0.363
LMR	5.85(4.82–6.85)	5.89(5.01–7.37)	0.417
PDW (%)	11.40(10.50–13.00)	11.70(10.73–13.35)	0.157
MPV (fl)	10.20(9.50–11.10)	10.40(9.73–11.00)	0.239
IL-1β (pg/ml)	9.83(8.42–11.28)	14.36(12.34–16.15)	**< 0.001**
IL-6 (pg/ml)	6.92(5.88–7.59)	8.95(8.31–10.12)	**< 0.001**
IL-8 (pg/ml)	1.00(0.82–1.21)	1.49(1.28–1.75)	**< 0.001**
IL-12 (pg/ml)	8.20(7.35–8.94)	10.46(9.52–11.31)	**< 0.001**
IL-17 (pg/ml)	32.45(28.33–37.70)	52.53(46.11–59.10)	**< 0.001**
IL-21 (pg/ml)	46.04(38.84–50.45)	62.02(55.01–67.84)	**< 0.001**
IL-32 (pg/ml)	18.41(15.70–20.01)	22.89(20.18–25.96)	**< 0.001**
TNF-α (pg/ml)	10.07(8.92–11.42)	13.88(12.22–15.54)	**< 0.001**

Data are expressed medians (IQR).

The bold values mean the *P*-values with statistically significant (*P* < 0.05).

NLR, neutrophil to lymphocyte ratio; PLR, platelet to lymphocyte ratio; LMR, lymphocyte to monocyte ratio; PDW, platelet distribution width; MPV, mean platelet volume; IL-1β, interleukin 1β; IL-6, interleukin 6; IL-8, interleukin 8; IL-12, interleukin 12; IL-17, interleukin 17; IL-21, interleukin 21; IL-32, interleukin 32; TNF-α, tumor necrosis factor-α.

### Correlation between inflammatory markers and other biochemical parameters in obese children

**Figure 1:**
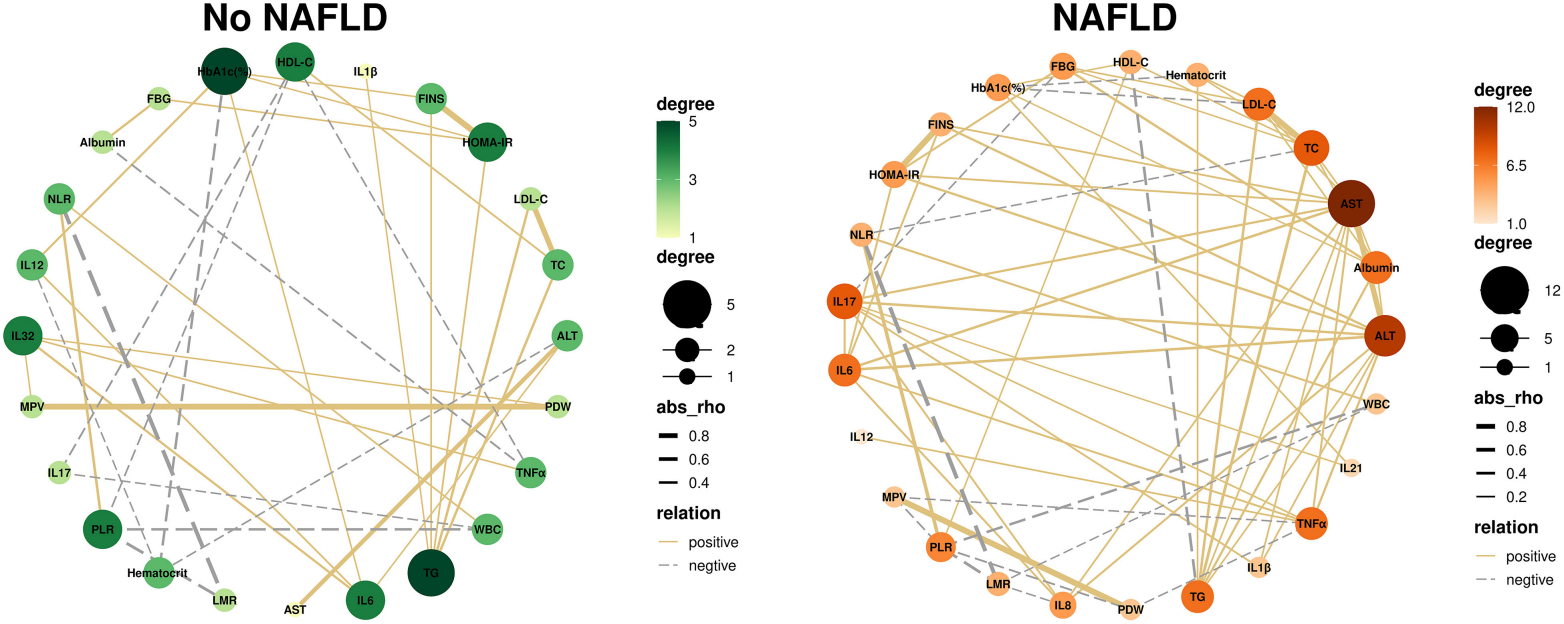
NAFLD leads to changes in the correlation between inflammatory markers and biochemical parameters. Correlation network analysis was performed on inflammatory markers, plotted against indicators that were significantly correlated (*P* < 0.05). Each circle represents a different indicator, the size and color of the circles represent the number of correlations involved for each indicator. The thickness of the line represents the rho value. The solid line represents a positive correlation, while the dashed line represents a negative correlation. TG, triglycerides; TC, total cholesterol; FINS, fasting insulin; HbA1c, glycosylated hemoglobin; HOMA-IR, homeostasis model assessment of insulin resistance; HDL-C, high-density lipoprotein cholesterol; LDL-C, low-density lipoprotein cholesterol; AST, aspartate aminotransferase; ALT, alanine aminotransferase; WBC, white blood cell count; PDW, platelet distribution width; MPV, mean platelet volume; WHR, waist circumference to hip circumference ratio; NLR, neutrophil to lymphocyte ratio; PLR, platelet to lymphocyte ratio; LMR, lymphocyte to monocyte ratio; IL-1β, interleukin-1β; IL-6, interleukin 6; IL-8, interleukin 8; IL-12, interleukin 12; IL-17, interleukin 17; IL-21, interleukin 21; IL-32, interleukin 32; TNF-α, tumor necrosis factor-α.

To determine the dynamic relationship and intensity between inflammatory markers and other biochemical parameters in different populations, we employed a network analysis using the Spearman's correlation matrix. The results showed that the networks in different groups differed in the correlations between inflammatory markers and other biochemical parameters. Most correlations in both groups were positive, meaning that an increase in the level of one specific marker was followed by increased levels of other biochemical parameters. Significantly, the network density was higher in the group of obese children with NAFLD (Figure 1).

We observed that inflammatory markers were significantly correlated with different biochemical parameters in the networks. In the group of obese children without NAFLD (i.e., the simple obesity group), we found that inflammatory markers IL-6 and IL-32 exhibited the highest number of significant correlations. IL-6 exhibited positive interactions with ALT, HbA1c, IL-12, and IL-32. And IL-32 exhibited positive interactions with MPV, PDW, IL-6, and TNF-α. While in the group of obese children with NAFLD, IL-17 was the most connected inflammatory marker in the networks, which was positively correlated with AST, ALT, IL-1β, IL-6, IL-8, IL-21, and TNF-α and was negatively correlated with FBG (Figure 1; Supplementary Tables 2, 3).

### Inflammatory markers associated with NAFLD

In univariable logistic regression analysis, we found IL-1β, IL-6, IL-8, IL-12, IL-17, IL-21, IL-32, and TNF-α were significantly associated with NAFLD [odds ratio (OR) = 1.13, 95% confidence interval (CI): 1.09–1.16, *P* < 0.001; OR = 1.25, 95% CI: 1.18–1.33, *P* < 0.001; OR = 2.39, 95% CI: 1.91–3.00, *P* < 0.001; OR = 1.17, 95% CI: 1.12–1.22, *P* < 0.001; OR = 1.04, 95% CI: 1.03–1.06, *P* < 0.001; OR = 1.04, 95% CI: 1.03–1.06, *P* < 0.001, OR = 1.06, 95% CI: 1.04–1.08, *P* < 0.001; OR = 1.14, 95% CI: 1.10–1.18, *P* < 0.001, respectively). Moreover, we found IL-1β, IL-6, IL-8, IL-12, IL-17, IL-21, IL-32, and TNF-α were significantly associated with NAFL, and IL-1β, IL-6, IL-8, IL-12, IL-17, and TNF-α were significantly associated with NASH. However, no significant associations were found for NLR, PLR, LMR, PDW, and MPV with NAFLD (all *P* > 0.05) (Table 3; Supplementary Table 4).

**Table 3:** Univariable analysis for inflammatory markers associated with NAFLD.

**Inflammatory markers (per 0.1 U)**	**OR**	**95%CI**	***P*-value**
NLR	0.99	0.95–1.03	0.620
PLR	1.00	0.99–1.00	0.314
LMR	1.00	0.99–1.01	0.502
PDW (%)	1.01	1.00–1.02	0.112
MPV (fl)	1.01	1.00–1.04	0.313
IL-1β (pg/ml)	1.13	1.09–1.16	**< 0.001**
IL-6 (pg/ml)	1.25	1.18–1.33	**< 0.001**
IL-8 (pg/ml)	2.39	1.91–3.00	**< 0.001**
IL-12 (pg/ml)	1.17	1.12–1.22	**< 0.001**
IL-17 (pg/ml)	1.04	1.03–1.06	**< 0.001**
IL-21 (pg/ml)	1.04	1.03–1.06	**< 0.001**
IL-32 (pg/ml)	1.06	1.04–1.08	**< 0.001**
TNF-α (pg/ml)	1.14	1.10–1.18	**< 0.001**

The bold values mean the *P*-values with statistically significant (*P* < 0.05).

NLR, neutrophil to lymphocyte ratio; PLR, platelet to lymphocyte ratio; LMR, lymphocyte to monocyte ratio; PDW, platelet distribution width; MPV, mean platelet volume; IL-1β, interleukin 1β; IL-6, interleukin 6; IL-8, interleukin 8; IL-12, interleukin 12; IL-17, interleukin 17; IL-21, interleukin 21; IL-32, interleukin 32; TNF-α, tumor necrosis factor-α.

Multivariable logistic regression analysis was conducted for the statistically significant factors above. After adjusting for sex, age, BMI, TG, HDL-C, AST, ALT, HbA1c, and HOMA-IR, we found significant associations for IL-1β (OR = 1.16, 95% CI: 1.04–1.29, *P* = 0.007), IL-6 (OR = 1.27, 95% CI: 1.07–1.50, *P* = 0.006), and IL-17 (OR = 1.04, 95% CI: 1.02–1.06, *P* = 0.001) with NAFLD (Table 4). Furthermore, we found significant associations for IL-1β, IL-6, and IL-17 with NAFL, and IL-1β, IL-6, IL-17, and TNF-α were significantly associated with NASH (Supplementary Table 5).

**Table 4:** Multivariable analysis for inflammatory markers associated with NAFLD.

**Inflammatory markers (per 0.1 U)**	OR*	**95%CI**	***P*-value**
IL-1β (pg/ml)	1.15	1.04–1.27	**0.007**
IL-6 (pg/ml)	1.28	1.07–1.53	**0.007**
IL-17 (pg/ml)	1.04	1.02–1.07	**0.001**

The bold values mean the *P*-values with statistically significant (*P* < 0.05). ^*^All ORs adjusted for sex, age, BMI, TG, HDL-C, AST, ALT, HbA1c, and HOMA-IR. IL-1β, interleukin 1β; IL-6, interleukin 6; IL-17, interleukin 17.

### Evaluation of inflammatory cytokines as biomarkers of NAFLD

**Figure 2:**
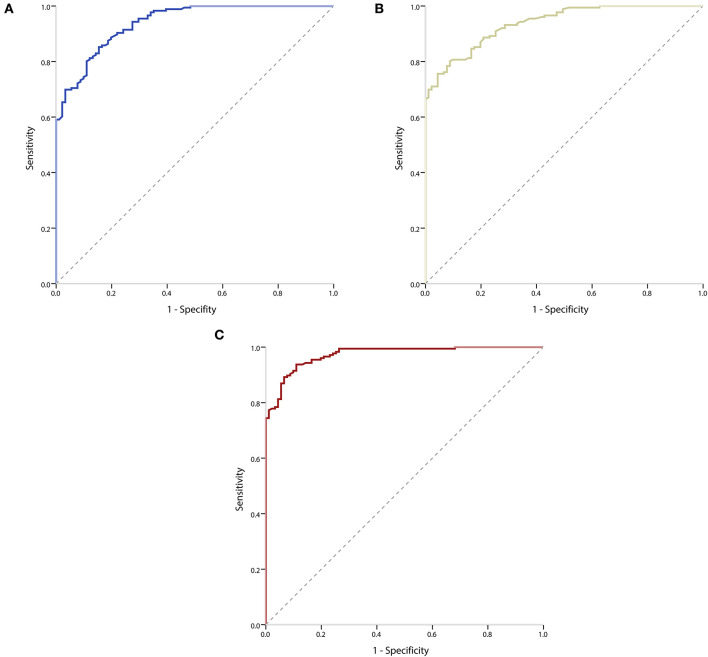
Receiver operating characteristic (ROC) curves of inflammatory markers to distinguish obese children with or without NAFLD. **(A)** ROC curve of IL-1β to distinguish obese children with or without NAFLD. **(B)** ROC curve of IL-6 to distinguish obese children with or without NAFLD. **(C)** ROC curve of IL-17 to distinguish obese children with or without NAFLD.

We used the ROC curve analysis to evaluate the accuracy of potential biomarkers to distinguish obese children with NAFLD from simple obese children. The area under curves (AUCs) of IL-1β, IL-6, and IL-17 to discriminate obese children with or without NAFLD were 0.94 (95% CI: 0.91–0.97, *P* < 0.001) (Figure 2A), 0.94 (95% CI: 0.91–0.96, *P* < 0.001) (Figure 2B), and 0.97 (95% CI: 0.96–0.99, *P* < 0.001) (Figure 2C), respectively. It was observed that three inflammatory markers had good diagnostic accuracy and the AUC of IL-17 was larger than IL-1β and IL-6. The cut-off value for IL-17 was 40.03 pg/ml, with a specificity of 93.8% and a sensitivity of 89.0%. The cut-off value for IL-1β was 11.74 pg/ml, with a specificity of 85.2% and a sensitivity of 84.6%. And the cut-off value for IL-6 was 8.10 pg/ml, with a specificity of 80.1% and a sensitivity of 91.2%.

## Discussion

In this study, we explored the relationship between inflammatory markers and NAFLD in obese children. We identified several biomarkers independently associated with NAFLD after adjustment for sex, age, BMI, TG, HDL-C, AST, ALT, HbA1c, and HOMA-IR. Our results indicated that increased IL-1β, IL-6, and IL-17 levels were significantly associated with NAFLD.

We found that plasma IL-1β, IL-6, IL-8, IL-12, IL-17, IL-21, IL-32, and TNF-α levels were elevated in obese children with NAFLD compared to subjects with simple obesity. These inflammatory markers levels were also elevated in obese children with NAFL and NASH. Furthermore, the levels of inflammatory markers were higher in obese children with NASH than in obese children with NAFL. These results were consistent with the findings of previous studies (20, 21), suggesting a trend of change in levels of inflammatory markers from simple obesity to NAFLD. Furthermore, we found that obese children with NAFLD exhibited distinct biochemical characteristics through correlation analysis, with closer correlations between biochemical parameters and inflammatory markers. Interestingly, obese children with NAFLD were marked with the presence of more positive correlations for inflammatory markers. These results may provide context for the potential role of inflammatory markers in NAFLD. Therefore, we hypothesize that NAFLD is characterized by a hepatic inflammatory environment leading to consistent changes in concentrations of systemic biochemical parameters.

Numerous studies have demonstrated that inflammatory cytokines played an essential role in the development of NAFLD through the activation of various inflammatory pathways that interfere with insulin signaling (31, 32), such as IL-1β, IL-6, and IL-17. Pro-inflammatory cytokines, including IL-1β and IL-6, were secreted by adipose tissue (33), which are involved in the inhibitor kappa B kinase beta/nuclear factor kappa B (IKK/NF-κB) pathway and the c-Jun N-terminal kinase/activator protein 1 (JNK/AP1) pathway through the activation of intracellular kinases (33, 34). Hepatic steatosis can increase the transcription factor NF-κβ signaling by activating upstream. The activation of NF-κβ induces the production of inflammatory cytokines, which helps recruit and activate Kupffer cells to mediate the inflammatory response (35, 36). IL-1β could activate Kupffer cells and promote the conversion of hepatic stellate cells to myofibroblasts, further aggravating the inflammatory response, and causing liver injury and hepatic fibrosis (37). IL-6 is a multi-biologically active cytokine that involves in insulin signaling and regulates acute phase responses and chronic inflammation (38, 39). Many pieces of evidence indicated positive associations for IL-1β and IL-6 with NAFLD (40–43).

Compared to other inflammatory cytokines, there were relatively few studies on IL-17 in NAFLD in obese children. IL-17 is a pro-inflammatory cytokine produced mainly by natural killer cells, natural killer T cells, and Th17 cells (44). Overexpression of IL-17 further increases lipid uptake by hepatocytes, exacerbating damage and making them susceptible to cell death and disease progression (45). IL-17 could promote the expression of other inflammatory cytokines, various inflammatory cytokines interacted together and collectively inhibited insulin signaling (31, 46). Similarly, a previous study found that Th17 cells and IL-17 were associated with hepatic steatosis and pro-inflammatory responses production in NAFLD (47). Furthermore, a study that also evaluated the relationship between cytokines and NAFLD in children confirmed our findings that plasma inflammatory cytokines were significantly associated with NAFLD (21). However, this study did not assess the association of IL-12, IL-17, IL-21, and IL-32 with NAFLD and did not include control subjects. Besides, recent studies have revealed a close link between the silent information regulator sirtuin 1 (SIRT1) and inflammation, and alterations in SIRT1 expression and activity have been associated with NAFLD (48–50). SIRT1 may be involved in the regulation of inflammatory cytokines in NAFLD and may be a key point in the progression of NAFLD. To sum up, we suggested that inflammatory cytokines were involved in the development of NAFLD and may contribute to its progression, facilitating the transition from NAFL to NASH.

In this study, we also developed prediction models using circulating levels of IL-1β, IL-6, and IL-17 as biomarkers to distinguish obese children with NAFLD from simple obese children. The spectrum of liver pathologies in NAFLD may lead to fibrosis, which progresses to advanced liver fibrosis, cirrhosis, hepatocellular carcinoma, and liver-related morbidity and mortality (51). It is significant to distinguish obese children with NAFLD from simple obese children for early treatment before the disease progresses to a more advanced stage. The results showed that the AUCs of IL-1β, IL-6, and IL-17 to discriminate obese children with or without NAFLD were 0.94, 0.94, and 0.97, respectively. This suggested that IL-1β, IL-6, and IL-17 had good diagnostic accuracy and the discriminative ability of IL-17 was superior to that of IL-1β and IL-6. Thus, these inflammatory cytokines could be considered non-invasive markers for differentiating NAFLD.

It should be mentioned that we did not find any association for NLR, PLR, LMR, PDW, and MPV with NAFLD. To our knowledge, this study was the first to evaluate associations for PLR and LMR with NAFLD in the children population. Some studies have shown that three novel compound inflammatory ratios, including NLR, PLR, and MPV, had higher predictive power than traditional inflammatory cytokines (52). And some studies have shown that PDW and MPV were widely used as biomarkers of platelet activation and function (24, 53). Unfortunately, the results obtained showed that they were not good non-invasive biomarkers of NAFLD in obese children. The main reason for these results may be the limited number of participants. Besides, negative results may be closely related to the multiple characteristics of these inflammatory markers involved in the development of NAFLD in a population of obese children. Of note, in this study, we found that inflammatory cytokines remained more predictive than other possible biomarkers.

This study has some strengths. First, a large number and well-characterized participants. Second, the number of inflammatory markers evaluated. Third, we used ROC curve analysis to assess the discriminative ability of inflammatory cytokines for NAFLD. Some potential limitations should also be considered. First, participants were recruited from one hospital which may have limited representation, and further multi-center studies are needed to validate our results, which could provide more credibility. Second, validation of NAFLD diagnosis with histological methods was not conducted in this study, as non-invasive assessments were more feasible in clinical practice. Thus, more studies with histological methods are needed in the future to confirm our results, including NAFL and NASH. Third, other inflammatory markers are also important and have the potential to be biomarkers of NAFLD, and more studies are needed in the future to explore their association with NAFLD in obese children. Fourth, our study was limited by the case-control study design, and we could not elucidate the causal relationship between inflammatory markers and NAFLD.

## Conclusion

Our study explored the relationship between inflammatory markers and NAFLD. We found that increased IL-1β, IL-6, and IL-17 levels were significantly associated with NAFLD, and they had good diagnostic accuracy to distinguish obese children with NAFLD from simple obese children. These plasma inflammatory cytokines may serve as non-invasive markers to improve the ability to determine the development of NAFLD and potentially identify additional avenues for therapeutic intervention.

## Data availability statement

The original contributions presented in the study are included in the article/Supplementary material, further inquiries can be directed to the corresponding author.

## Ethics statement

The studies involving human participants were reviewed and approved by Ethics Committee of Xiangya School of Public Health: HCHLL-2019-12. Written informed consent to participate in this study was provided by the participants' legal guardian/next of kin.

## Author contributions

YD, JLu, XP, and ML designed the study. YD wrote the manuscript with support from other authors. XP and JW contributed to the statistical analysis. XX and JLi contributed to preparing the tables and figures. ML, YD, and JLu reviewed and revised the manuscript. All authors approved the final version of the article, including the authorship list.

## Funding

This work was supported by the National Natural Science Foundation of China (81872641) and the Natural Science Foundation of Hunan Province (2021JJ30901 and 2022JJ40668).

## Conflict of interest

The authors declare that the research was conducted in the absence of any commercial or financial relationships that could be construed as a potential conflict of interest.

## Publisher's note

All claims expressed in this article are solely those of the authors and do not necessarily represent those of their affiliated organizations, or those of the publisher, the editors and the reviewers. Any product that may be evaluated in this article, or claim that may be made by its manufacturer, is not guaranteed or endorsed by the publisher.

## References

[1] Pan XF Wang L Pan A. Epidemiology and determinants of obesity in China. Lancet Diabetes Endocrinol. (2021) 9:373–92. 10.1016/S2213-8587(21)00045-0 34022156

[2] Li B Adab P Cheng KK. The role of grandparents in childhood obesity in China-evidence from a mixed methods study. Int J Behav Nutr Phys Act. (2015) 12:91. 10.1186/s12966-015-0251-z 26122955 PMC4507318

[3] Chang Y Jung HS Cho J Zhang Y Yun KE Lazo M. Metabolically healthy obesity and the development of nonalcoholic fatty liver disease. Am J Gastroenterol. (2016) 111:1133–40. 10.1038/ajg.2016.178 27185080

[4] Shaunak M Byrne CD Davis N Afolabi P Faust SN Davies JH. Non-alcoholic fatty liver disease and childhood obesity. Arch Dis Child. (2021) 106:3–8. 10.1136/archdischild-2019-318063 32409495

[5] Song P Yu J Wang M Chang X Wang J An L. Prevalence and correlates of suspected nonalcoholic fatty liver disease in chinese children. Int J Environ Res Public Health. (2017) 14:465. 10.3390/ijerph14050465 28448433 PMC5451916

[6] Mann JP Valenti L Scorletti E Byrne CD Nobili V. Nonalcoholic fatty liver disease in children. Semin Liver Dis. (2018) 38:1–13. 10.1055/s-0038-1627456 29471561

[7] Powell EE Wong VW Rinella M. Non-alcoholic fatty liver disease. Lancet. (2021) 397:2212–24. 10.1016/S0140-6736(20)32511-3 33894145

[8] Sutti S Albano E. Adaptive immunity: an emerging player in the progression of NAFLD. Nat Rev Gastroenterol Hepatol. (2020) 17:81–92. 10.1038/s41575-019-0210-2 31605031 PMC7222953

[9] Rinella ME. Nonalcoholic fatty liver disease: a systematic review. JAMA. (2015) 313:2263–73. 10.1001/jama.2015.5370 26057287

[10] Pierantonelli I Svegliati-Baroni G. Nonalcoholic fatty liver disease: basic pathogenetic mechanisms in the progression from NAFLD to NASH. Transplantation. (2019) 103:e1–e13. 10.1097/TP.0000000000002480 30300287

[11] Friedman SL Neuschwander-Tetri BA Rinella M Sanyal AJ. Mechanisms of NAFLD development and therapeutic strategies. Nat Med. (2018) 24:908–22. 10.1038/s41591-018-0104-9 29967350 PMC6553468

[12] Peng C Stewart AG Woodman OL Ritchie RH Qin CX. Non-alcoholic steatohepatitis: a review of its mechanism, models and medical treatments. Front Pharmacol. (2020) 11:603926. 10.3389/fphar.2020.603926 33343375 PMC7745178

[13] Kitade H Chen G Ni Y Ota T. Nonalcoholic fatty liver disease and insulin resistance: new insights and potential new treatments. Nutrients. (2017) 9:387. 10.3390/nu9040387 28420094 PMC5409726

[14] Perla FM Prelati M Lavorato M Visicchio D Anania C. The role of lipid and lipoprotein metabolism in non-alcoholic fatty liver disease. Children. (2017) 4:46. 10.3390/children4060046 28587303 PMC5483621

[15] Schuster S Cabrera D Arrese M Feldstein AE. Triggering and resolution of inflammation in NASH. Nat Rev Gastroenterol Hepatol. (2018) 15:349–64. 10.1038/s41575-018-0009-6 29740166

[16] Umano GR Martino M Santoro N. The association between pediatric NAFLD and common genetic variants. Children. (2017) 4:49. 10.3390/children4060049 28629152 PMC5483624

[17] Takahashi Y Fukusato T. Pediatric nonalcoholic fatty liver disease: overview with emphasis on histology. World J Gastroenterol. (2010) 16:5280–5. 10.3748/wjg.v16.i42.5280 21072890 PMC2980676

[18] Malehmir M Pfister D Gallage S Szydlowska M Inverso D Kotsiliti E. Platelet GPIbα is a mediator and potential interventional target for NASH and subsequent liver cancer. Nat Med. (2019) 25:641–55. 10.1038/s41591-019-0379-5 30936549 PMC12452109

[19] Zhang TS Qin HL Wang T Li HT Li H Xia SH. Global publication trends and research hotspots of nonalcoholic fatty liver disease: a bibliometric analysis and systematic review. Springerplus. (2015) 4:776. 10.1186/s40064-015-1542-1 26697286 PMC4678134

[20] Ajmera V Perito ER Bass NM Terrault NA Yates KP Gill R. Novel plasma biomarkers associated with liver disease severity in adults with nonalcoholic fatty liver disease. Hepatology. (2017) 65:65–77. 10.1002/hep.28776 27532276 PMC5191932

[21] Perito ER Ajmera V Bass NM Rosenthal P Lavine JE Schwimmer JB. Association between cytokines and liver histology in children with nonalcoholic fatty liver disease. Hepatol Commun. (2017) 1:609–22. 10.1002/hep4.1068 29130075 PMC5679472

[22] Gong P Liu Y Gong Y Chen G Zhang X Wang S. The association of neutrophil to lymphocyte ratio, platelet to lymphocyte ratio, and lymphocyte to monocyte ratio with post-thrombolysis early neurological outcomes in patients with acute ischemic stroke. J Neuroinflammation. (2021) 18:51. 10.1186/s12974-021-02090-6 33610168 PMC7896410

[23] Vukicevic P Klisic A Neskovic V Babic L Mikic A Bogavac-Stanojevic N. New markers of platelet activation and reactivity and oxidative stress parameters in patients undergoing coronary artery bypass grafting. Oxid Med Cell Longev. (2021) 2021:8915253. 10.1155/2021/8915253 34257821 PMC8257340

[24] Khoury T Mari A Nseir W Kadah A Sbeit W Mahamid M. Neutrophil-to-lymphocyte ratio is independently associated with inflammatory activity and fibrosis grade in nonalcoholic fatty liver disease. Eur J Gastroenterol Hepatol. (2019) 31:1110–5. 10.1097/MEG.0000000000001393 30888972

[25] Chauhan A Adams DH Watson SP Lalor PF. Platelets: No longer bystanders in liver disease. Hepatology. (2016) 64:1774–84. 10.1002/hep.28526 26934463 PMC5082495

[26] Hou X Yin S Ren R Liu S Yong L Liu Y. Myeloid-cell-specific IL-6 signaling promotes MicroRNA-223-enriched exosome production to attenuate NAFLD-associated fibrosis. Hepatology. (2021) 74:116–32. 10.1002/hep.31658 33236445 PMC8141545

[27] du Plessis J van Pelt J Korf H Mathieu C van der Schueren B Lannoo M. Association of adipose tissue inflammation with histologic severity of nonalcoholic fatty liver disease. Gastroenterology. (2015) 149:635–48.e14. 10.1053/j.gastro.2015.05.044 26028579

[28] Cabré N Luciano-Mateo F Fernández-Arroyo S Baiges-Gayà G Hernández-Aguilera A Fibla M. Laparoscopic sleeve gastrectomy reverses non-alcoholic fatty liver disease modulating oxidative stress and inflammation. Metabolism. (2019) 99:81–9. 10.1016/j.metabol.2019.07.002 31279739

[29] Viglino D Jullian-Desayes I Minoves M Aron-Wisnewsky J Leroy V Zarski JP. Nonalcoholic fatty liver disease in chronic obstructive pulmonary disease. Eur Respir J. (2017) 49:1601923. 10.1183/13993003.01923-2016 28596431

[30] Zhou X Fu J. Expert consensus on the diagnosis and treatment of non-alcoholic fatty liver disease in children. Chin J Pract Pediatrics. (2018) 33:487–92.

[31] Khan RS Bril F Cusi K Newsome PN. Modulation of insulin resistance in nonalcoholic fatty liver disease. Hepatology. (2019) 70:711–24. 10.1002/hep.30429 30556145

[32] Asrih M Jornayvaz FR. Metabolic syndrome and nonalcoholic fatty liver disease: is insulin resistance the link? Mol Cell Endocrinol. (2015) 418:55–65. 10.1016/j.mce.2015.02.018 25724480

[33] Mu W Cheng XF Liu Y Lv QZ Liu GL Zhang JG. Potential nexus of non-alcoholic fatty liver disease and type 2 diabetes mellitus: insulin resistance between hepatic and peripheral tissues. Front Pharmacol. (2018) 9:1566. 10.3389/fphar.2018.01566 30692925 PMC6339917

[34] Uemura H Katsuura-Kamano S Yamaguchi M Bahari T Ishizu M Fujioka M. Relationships of serum high-sensitivity C-reactive protein and body size with insulin resistance in a Japanese cohort. PLoS ONE. (2017) 12:e0178672. 10.1371/journal.pone.0178672 28575103 PMC5456096

[35] Nagai H Matsumaru K Feng G Kaplowitz N. Reduced glutathione depletion causes necrosis and sensitization to tumor necrosis factor-alpha-induced apoptosis in cultured mouse hepatocytes. Hepatology. (2002) 36:55–64. 10.1053/jhep.2002.33995 12085349

[36] Huang J Hao C Li Z Wang L Jiang J Tang W. NRF2-617 C/A polymorphism impacts proinflammatory cytokine levels, survival, and transplant-related mortality after hematopoietic stem cell transplantation in adult patients receiving busulfan-based conditioning regimens. Front Pharmacol. (2020) 11:563321. 10.3389/fphar.2020.563321 33384597 PMC7770105

[37] Mollica MP Lionetti L Putti R Cavaliere G Gaita M Barletta A. From chronic overfeeding to hepatic injury: role of endoplasmic reticulum stress and inflammation. Nutr Metab Cardiovasc Dis. (2011) 21:222–30. 10.1016/j.numecd.2010.10.012 21277757

[38] Cobbina E Akhlaghi F. Non-alcoholic fatty liver disease (NAFLD)-pathogenesis, classification, and effect on drug metabolizing enzymes and transporters. Drug Metab Rev. (2017) 49:197–211. 10.1080/03602532.2017.1293683 28303724 PMC5576152

[39] Lima-Cabello E García-Mediavilla MV Miquilena-Colina ME Vargas-Castrillón J Lozano-Rodríguez T Fernández-Bermejo M. Enhanced expression of pro-inflammatory mediators and liver X-receptor-regulated lipogenic genes in non-alcoholic fatty liver disease and hepatitis C. Clin Sci. (2011) 120:239–50. 10.1042/CS20100387 20929443

[40] Gao B Tsukamoto H. Inflammation in alcoholic and nonalcoholic fatty liver disease: friend or foe? Gastroenterology. (2016) 150:1704–9. 10.1053/j.gastro.2016.01.025 26826669 PMC4887345

[41] Ajmal MR Yaccha M Malik MA Rabbani MU Ahmad I Isalm N. Prevalence of nonalcoholic fatty liver disease (NAFLD) in patients of cardiovascular diseases and its association with hs-CRP and TNF-α. Indian Heart J. (2014) 66:574–9. 10.1016/j.ihj.2014.08.006 25634387 PMC4310973

[42] Seo YY Cho YK Bae JC Seo MH Park SE Rhee EJ. Tumor necrosis factor-alpha as a predictor for the development of nonalcoholic fatty liver disease: a 4-year follow-up study. Endocrinol Metab. (2013) 28:41–5. 10.3803/EnM.2013.28.1.41 24396649 PMC3811803

[43] Duan Y Pan X Luo J Xiao X Li J Bestman PL. Association of inflammatory cytokines with non-alcoholic fatty liver disease. Front Immunol. (2022) 13:880298. 10.3389/fimmu.2022.880298 35603224 PMC9122097

[44] Giles DA Moreno-Fernandez ME Divanovic S. IL-17 axis driven inflammation in non-alcoholic fatty liver disease progression. Curr Drug Targets. (2015) 16:1315–23. 10.2174/1389450116666150531153627 26028039 PMC4929857

[45] Berlanga A Guiu-Jurado E Porras JA Auguet T. Molecular pathways in non-alcoholic fatty liver disease. Clin Exp Gastroenterol. (2014) 7:221–39. 10.2147/CEG.S62831 25045276 PMC4094580

[46] Ruiz de Morales JMG Puig L Daudén E Cañete JD Pablos JL Martín AO. Critical role of interleukin (IL)-17 in inflammatory and immune disorders: An updated review of the evidence focusing in controversies. Autoimmun Rev. (2020) 19:102429. 10.1016/j.autrev.2019.102429 31734402

[47] Tang Y Bian Z Zhao L Liu Y Liang S Wang Q. Interleukin-17 exacerbates hepatic steatosis and inflammation in non-alcoholic fatty liver disease. Clin Exp Immunol. (2011) 166:281–90. 10.1111/j.1365-2249.2011.04471.x 21985374 PMC3219903

[48] Martins IJ. Insulin therapy and autoimmune disease with relevance to non alchoholic fatty liver disease. In: Gad EH, editor. Nonalcoholic Fatty Liver Disease-An Update. London: IntechOpen (2018).

[49] Martins IJ. Genomic medicine and endocrine autoimmunity as key to mitochondrial disease. Global Journal of Endocrinological Metabolism. (2018) 2:1–3. 10.31031/GJEM.2018.02.00053426799652

[50] Yang Y Liu Y Wang Y Chao Y Zhang J Jia Y. Regulation of SIRT1 and its roles in inflammation. Front Immunol. (2022) 13:831168. 10.3389/fimmu.2022.831168 35359990 PMC8962665

[51] Loomba R Friedman SL Shulman GI. Mechanisms and disease consequences of nonalcoholic fatty liver disease. Cell. (2021) 184:2537–64. 10.1016/j.cell.2021.04.015 33989548 PMC12168897

[52] Curbelo J Luquero Bueno S Galván-Román JM Ortega-Gómez M Rajas O Fernández-Jiménez G. Inflammation biomarkers in blood as mortality predictors in community-acquired pneumonia admitted patients: importance of comparison with neutrophil count percentage or neutrophil-lymphocyte ratio. PLoS ONE. (2017) 12:e0173947. 10.1371/journal.pone.0173947 28301543 PMC5354424

[53] Abeles RD Mullish BH Forlano R Kimhofer T Adler M Tzallas A. Derivation and validation of a cardiovascular risk score for prediction of major acute cardiovascular events in non-alcoholic fatty liver disease; the importance of an elevated mean platelet volume. Aliment Pharmacol Ther. (2019) 49:1077–85. 10.1111/apt.15192 30836450 PMC6519040

