# Supplementary material for: Association between inflammatory markers and non-alcoholic fatty liver disease in obese children
Source: Front Public Health. 2022 Dec 1;10:991393. doi: 10.3389/fpubh.2022.991393 (PMC9751435; doi:10.3389/fpubh.2022.991393)
Supplement: Supplementary file 1 [file Table_1.DOCX]

**Supplementary 1**. Levels of inflammatory markers of the subgroup by NAFLD diagnosis

| Variables | Simple obesity (n=91) | NAFLD (n=176) | | *P*-value |
| --- | --- | --- | --- | --- |
|  |  | NAFL (n=110) | NASH (n=66) |  |
| NLR | 1.42(1.17-1.96) | 1.48(1.12-1.84) | 1.52(1.19-1.78) | 0.862 |
| PLR | 117.79(95.96-151.17) | 114.20(94.34-136.40) | 113.82(92.27-136.47) | 0.604 |
| LMR | 5.85(4.82-6.85) | 6.01(5.02-7.85) | 5.83(4.92-7.17) | 0.534 |
| PDW (%) | 11.40(10.50-13.00) | 11.70(10.70-13.00) | 11.80(10.80-13.65) | 0.328 |
| MPV (fl) | 10.20(9.50-11.10) | 10.37(9.80-10.90) | 10.40(9.68-11.10) | 0.454 |
| IL-1β (pg/mL) | 9.83(8.42-11.28) | 13.97(12.15-15.78) ^a^ | 15.31(13.36-17.06) ^bc^ | **<0.001** |
| IL-6 (pg/mL) | 6.92(5.88-7.59) | 8.62(7.84-9.41) ^a^ | 9.64(8.77-10.59) ^bc^ | **<0.001** |
| IL-8 (pg/mL) | 1.00(0.82-1.21) | 1.43(1.25-1.65) ^a^ | 1.69(1.39-1.90) ^bc^ | **<0.001** |
| IL-12 (pg/mL) | 8.20(7.35-8.94) | 10.42(9.34-11.13) ^a^ | 10.52(9.77-11.60) ^b^ | **<0.001** |
| IL-17 (pg/mL) | 32.45(28.33-37.70) | 49.05(41.96-57.38) ^a^ | 55.88(50.13-63.55) ^bc^ | **<0.001** |
| IL-21 (pg/mL) | 46.04(38.84-50.45) | 62.02(55.15-67.05) ^a^ | 61.82(54.06-68.71) ^b^ | **<0.001** |
| IL-32 (pg/mL) | 18.41(15.70-20.01) | 22.64(19.93-25.79) ^a^ | 23.19(20.20-26.31) ^b^ | **<0.001** |
| TNF-α (pg/mL) | 10.07(8.92-11.42) | 13.44(11.64-14.79) ^a^ | 14.84(13.19-17.14) ^bc^ | **<0.001** |

Data are expressed medians (IQR).

a: Significant differences between NAFL and simpe obesity (*P* < 0.05).

b: Significant differences between NASH and simple obesity (*P* < 0.05).

c: Significant differences between NASH and NAFL (*P* < 0.05).

The bold values mean the *P* values with statistically significant (*P* < 0.05).

Abbreviations: NLR, neutrophil to lymphocyte ratio; PLR, platelet to lymphocyte ratio; LMR, lymphocyte to monocyte ratio; PDW, platelet distribution width; MPV, mean platelet volume; IL-1β, interleukin 1β; IL-6, interleukin 6; IL-8, interleukin 8; IL-12, interleukin 12; IL-17, interleukin 17; IL-21, interleukin 21; IL-32, interleukin 32; TNF-α, tumor necrosis factor-α.

**Supplementary 4**. Univariable analysis for inflammatory markers associated with different stages of NAFLD

| Inflammatory markers | NAFL (vs. Simple obesity) | | NASH (vs. NAFL) | |
| --- | --- | --- | --- | --- |
|  | OR (95%CI)  per 0.1U | *P*值 | OR (95%CI)  per 0.1U | *P*值 |
| NLR | 0.99(0.95-1.04) | 0.734 | 0.99(0.94-1.05) | 0.802 |
| PLR | 1.00(0.99-1.00) | 0.465 | 1.00(0.99-1.00) | 0.646 |
| LMR | 1.00(0.99-1.01) | 0.802 | 0.99(0.97-1.01) | 0.205 |
| PDW (%) | 1.01(1.00-1.02) | 0.225 | 1.01(0.99-1.02) | 0.502 |
| MPV (fl) | 1.01(0.98-1.04) | 0.474 | 1.01(0.98-1.04) | 0.578 |
| IL-1β (pg/mL) | 1.12(1.09-1.16) | **<0.001** | 1.03(1.01-1.04) | **0.001** |
| IL-6 (pg/mL) | 1.23(1.16-1.31) | **<0.001** | 1.09(1.06-1.13) | **<0.001** |
| IL-8 (pg/mL) | 2.26(1.80-2.84) | **<0.001** | 1.31(1.16-1.48) | **<0.001** |
| IL-12 (pg/mL) | 1.16(1.11-1.20) | **<0.001** | 1.03(1.00-1.06) | **0.022** |
| IL-17 (pg/mL) | 1.04(1.03-1.05) | **<0.001** | 1.01(1.01-1.02) | **<0.001** |
| IL-21 (pg/mL) | 1.04(1.03-1.06) | **<0.001** | 1.00(0.99-1.00) | 0.809 |
| IL-32 (pg/mL) | 1.06(1.04-1.07) | **<0.001** | 1.01(0.99-1.02) | 0.182 |
| TNF-α (pg/mL) | 1.13(1.09-1.17) | **<0.001** | 1.04(1.03-1.06) | **<0.001** |

The bold values mean the *P* values with statistically significant (*P* < 0.05).

Abbreviations: NLR, neutrophil to lymphocyte ratio; PLR, platelet to lymphocyte ratio; LMR, lymphocyte to monocyte ratio; PDW, platelet distribution width; MPV, mean platelet volume; IL-1β, interleukin 1β; IL-6, interleukin 6; IL-8, interleukin 8; IL-12, interleukin 12; IL-17, interleukin 17; IL-21, interleukin 21; IL-32, interleukin 32; TNF-α, tumor necrosis factor-α.

**Supplementary 5**. Multivariable analysis for inflammatory markers associated with different stages of NAFLD

| Outcomes | Inflammatory markers | Odds Ratio* | OR (95%CI)  per 0.1U | *P*值 |
| --- | --- | --- | --- | --- |
| NAFL  (vs. Simple obesity) | IL-1β (pg/mL) | 1.16 | 1.04-1.29 | **0.007** |
|  | IL-6 (pg/mL) | 1.27 | 1.07-1.50 | **0.006** |
|  | IL-17 (pg/mL) | 1.04 | 1.02-1.06 | **0.001** |
| NASH  (vs. NAFL) | IL-1β (pg/mL) | 1.03 | 1.00-1.05 | **0.017** |
|  | IL-6 (pg/mL) | 1.08 | 1.03-1.12 | **0.001** |
|  | IL-8 (pg/mL) | 1.19 | 1.00-1.41 | 0.051 |
|  | IL-12 (pg/mL) | 1.01 | 0.98-1.05 | 0.483 |
|  | IL-17 (pg/mL) | 1.01 | 1.00-1.02 | **0.005** |
|  | IL-21 (pg/mL) | 1.00 | 1.00-1.01 | 0.736 |
|  | IL-32 (pg/mL) | 1.01 | 0.99-1.02 | 0.343 |
|  | TNF-α (pg/mL) | 1.04 | 1.02-1.06 | **0.001** |

*All Odds Ratios adjusted for sex, age, BMI, WHR, TG, HDL-C, HbA1c, and HOMA-IR.

The bold values mean the *P* values with statistically significant (*P* < 0.05).

Abbreviations: IL-1β, interleukin 1β; IL-6, interleukin 6; IL-8, interleukin 8; IL-12, interleukin 12; IL-17, interleukin 17; IL-21, interleukin 21; IL-32, interleukin 32; TNF-α, tumor necrosis factor-α.
